# Chromosome-Level Genome Assembly of *Ormosia henryi* Provides Insights into Evolutionary Resilience and Precision Conservation

**DOI:** 10.3390/plants15020180

**Published:** 2026-01-07

**Authors:** Xiaoming Tian, Bin Yuan, Cun Mou, Guangfeng Xiang, Lu Zhu, Gaofei Li, Chao Liu, Xiangpeng Li, Fuliang Hu, Hao Lv

**Affiliations:** 1 Hunan Botanical Garden, Changsha 410004, China; tianxiaoming1986@126.com (X.T.);; 2 College of Animal Sciences, Zhejiang University, Hangzhou 310058, China; yuan_bin322@zju.edu.cn

**Keywords:** *Ormosia henryi*, chromosome-level genome, comparative genomics, gene family expansion, conservation genetics, sustainable utilization

## Abstract

*Ormosia henryi*, a rare and endemic timber tree in China, possesses exceptional economic and ecological value, but it has experienced a critical decline in wild populations. We integrated PacBio HiFi and Hi-C technologies to generate a superior, chromosome-level genome assembly, establishing a more robust genetic foundation than existing draft sequences. The resulting assembly (2.64 Gb; Contig N50 = 39.17 Mb; and Scaffold N50 = 338.40 Mb) exhibits high continuity and completeness, effectively overcoming the assembly challenges associated with high heterozygosity (1.37%) and repetitive sequence content (83.89%). Comparative genomic analysis revealed that *O. henryi* diverged from *Lupinus albus* approximately 53.82 million years ago and underwent two independent whole-genome duplication events. The historical accumulation of evolutionary resilience is reflected in the significant expansion of 276 gene families enriched in photosynthesis and phenylpropanoid biosynthesis, alongside 122 genes under positive selection involved in DNA repair and proteostasis. These genomic signatures elucidate a stable genetic foundation. While wild populations have sharply declined in recent decades, this suggests that this status underscores the overwhelming impact of intense external anthropogenic pressures, such as overexploitation and habitat fragmentation, which may have overridden the species’ inherent adaptive capacity and slow life-history strategy. This high-quality genomic resource identifies key candidate loci, such as the *PIF1* helicase for growth regulation, and provides a critical framework for screening elite germplasm for population restoration. Consequently, this study establishes a theoretical and molecular basis for transitioning from fundamental research to the precision conservation and sustainable industrial application of this high-value woody species.

## 1. Introduction

**Figure 1:**
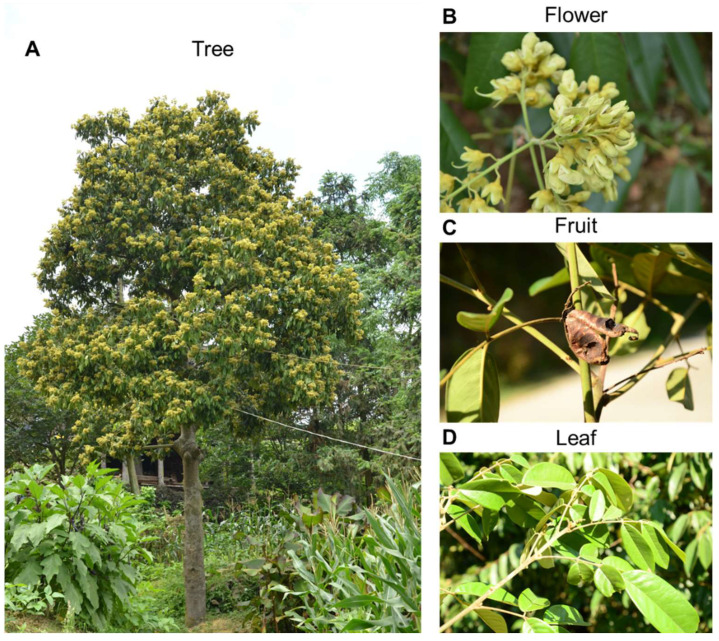
Tree and organs of *Ormosia henryi*: (**A**) tree; (**B**) flower; (**C**) fruit; (**D**) leaf.

*Ormosia henryi* (2n = 16; Figure 1), an endemic and precious tree species in China, belongs to the family Fabaceae [1]. It is highly regarded for its significant ecological, medicinal (e.g., its leaves, roots, stems, and seeds are used to treat ailments such as bruises and arthritis), cultural, and ornamental value. Historically, various parts of the tree, including its leaves, roots, stems, and seeds, have been utilized in traditional medicine to treat conditions such as bruises and arthritis. Its timber is characterized by a distinctive fragrance, dense texture, and high hardness, making it an ideal raw material for furniture and handicrafts with substantial economic value [2,3]. However, the exceptional value of its timber has led to persistent threats from illegal logging and overexploitation [4]. Wild populations of *O. henryi* have experienced a marked decline, a situation further exacerbated by inherent biological constraints, including a slow growth rate, low natural seed set, and germination difficulties due to a hard seed coat [5,6]. It is currently listed as a National Class II Protected Wild Plant in China [6].

It is essential to decipher its genetic architecture to ensure the sustainable utilization and effective conservation of this valuable resource. Recent advances have led to the publication of genome sequences for several *Ormosia* species, including *O. henryi*, *O. purpureiflora*, *O. emarginata*, *O. semicastrata*, and *O. boluoensis*. These studies have provided foundational insights into the genus, revealing common genomic features, including relatively large genome sizes, high repetitive-sequence content, and high levels of heterozygosity [6,7,8,9]. However, these studies have focused either on constructing genome sketches or on obtaining chromosome-level assemblies, but in-depth comparative genomics analyses have not been conducted. Therefore, little is known about how *Ormosia* species (especially *O. henryi*) evolve genetic mechanisms to adapt to specific environments at the genomic level.

In this study, we aimed to address these gaps by constructing a high-quality, chromosome-level reference genome for *O. henryi* using a combination of PacBio HiFi sequencing and Hi-C scaffolding technology. We investigated genome structure evolution, gene family dynamics, and key pathways subject to positive selection by integrating this new assembly with comparative genomic analyses involving nine Fabaceae species. Our analysis focused on elucidating the species’ evolutionary position, divergence timing, whole-genome duplication (WGD) events, and genomic collinearity. These findings are intended to provide core genomic resources and a theoretical framework to support the conservation, genetic improvement, and trait-directed breeding of *O. henryi*.

## 2. Results

### 2.1. High-Quality Chromosome-Scale Genome Assembly

**Figure 2:**
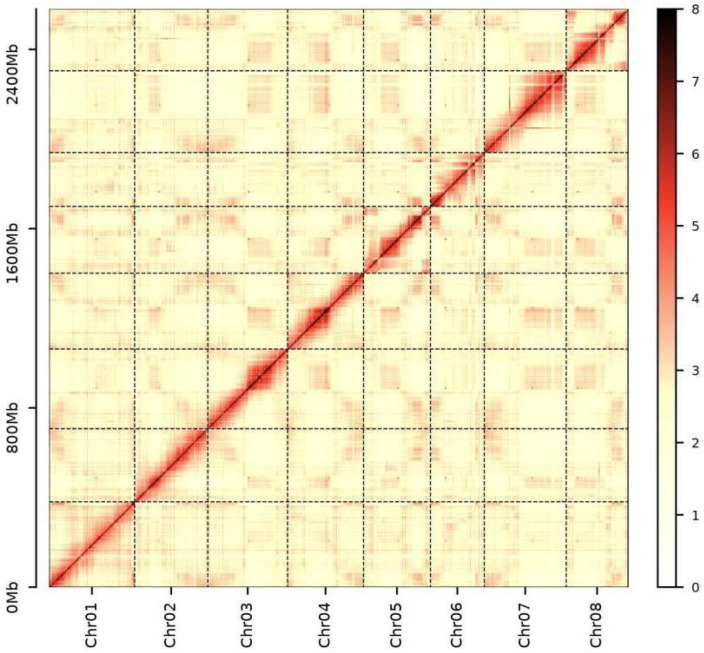
Hi-C heatmap for the genome assembly of *Ormosia henryi* (bin size = 900,000 b).

We successfully assembled a chromosome-level genome for *O. henryi* by integrating PacBio HiFi long-read sequencing (96.52 Gb; depth: 36.7×), Illumina short-read error correction, and Hi-C chromosome conformation capture technology (434.2 Gb of clean data; depth: 165.10×) (Table S2). The assembled genome size was 2.64 Gb, with a contig N50 of 39.17 Mb and a scaffold N50 of 338.40 Mb, representing the highest contiguity assembly among currently published *Ormosia* species (Table 1 and Table S3, and Figure S1). A total of 97.8% of the sequences (2,639,023,864 bp) were successfully anchored onto eight pseudochromosomes (Table S2). The Hi-C interaction map revealed distinct chromosome boundaries and clear intra-chromosomal interaction signals (Figure 2). The BUSCO assessment demonstrated a high assembly completeness of 98.3% (90.7% and 7.6% single-copy and duplicated genes, respectively), confirming the assembly’s completeness and reliability (Table 2).

### 2.2. Genomic Features and Gene Annotation

**Figure 3:**
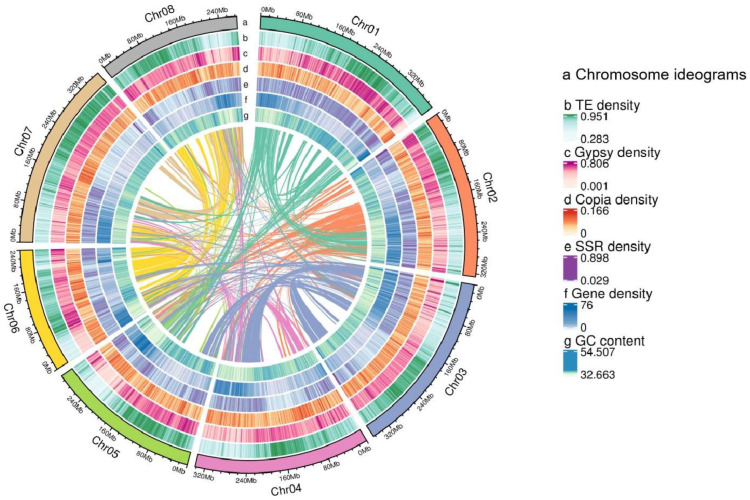
Genomic features of *Ormosia henryi*. The features are arranged in the order of chromosomes, GC content, gene density, SSR density, Copia density, Gypsy density, TE density, and syntenic blocks from outside to inside across the eight pseudochromosomes.

Repetitive sequences constitute 83.89% of the *O. henryi* genome, with transposable elements (TEs), tandem repeats (TRFs), long terminal repeat (LTR) retrotransposons, and DNA transposons accounting for 78.45%, 5.44%, 74.14%, and 3.90%, respectively (Figure 3 and Table S4). A total of 182,994 intact Copia and 631,036 intact Gypsy elements were identified among the LTR retrotransposons (Table S4). Non-coding RNAs (ncRNAs) represent 0.0005% of the genome, comprising ribosomal RNAs (rRNAs (9174)), transfer RNAs (tRNAs (2068)), and small RNAs (including miRNAs, snRNAs, and snoRNAs, totaling 2954 (Table S5)). Pseudogenes account for 0.21% of the genome, totaling 776, with a cumulative length of 5,649,925 bp and an average length of 7280.83 bp (Table S6).

Based on this genomic assembly, we predicted 39,017 protein-coding genes using integrated ab initio, homology-based, and transcriptome-supported approaches (Table S7). Functional annotation was achieved for 99.32% (38,753) of these genes in at least one public database (Table 3). Annotation revealed that 72.57%, 79.92%, and 77.79% of the genes matched entries in the SwissProt database; these were assigned Gene Ontology (GO) functional classifications (Figure S2) and were mapped to KEGG metabolic pathways (Figure S3). Finally, the BUSCO analysis showed that 98.57% of genes were identified during annotation, indicating that the annotation was complete and reliable (Table S8).

### 2.3. Comparative Genomics and Evolutionary Insights

**Figure 4:**
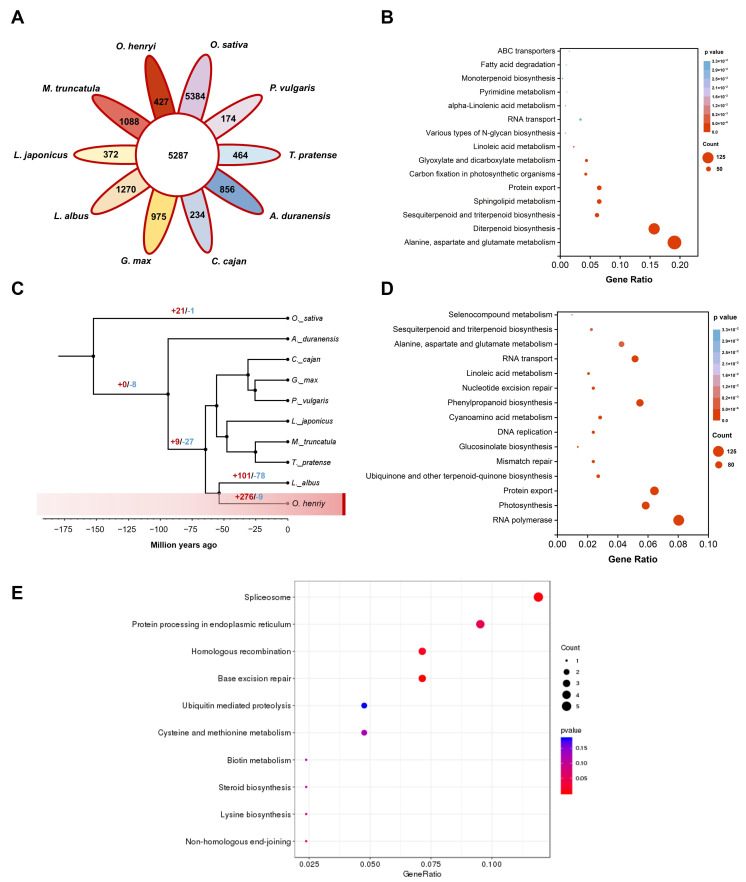
Comparative genomics and evolution. (**A**) Comparative analysis of gene families of *O. henryi* and nine widely distributed Fabaceae plants (*Trifolium pratense*, *Lupinus albus*, *Glycine max*, *Arachis duranensis*, *Medicago truncatula*, *Lotus japonicus*, *Cajanus cajan*, and *Phaseolus vulgaris*), with *Oryza sativa* as the outgroup; (**B**) KEGG enrichment of unique gene families to *O. henryi* compared with that of other plants in (**A**); (**C**) evolutionary tree of 10 plant species in (**A**), with *Oryza sativa* as the outgroup, numbers on the phylogenetic tree indicate the expanded (red) or contracted (blue) gene families compares to their ancestors; (**D**) KEGG enrichment of expanded gene families in *O. henryi*; and (**E**) KEGG enrichment of positively selected genes.

Comparative genomic analysis across nine Fabaceae species (Figure 4A) identified 37,523 gene families. Among these, 427 gene families (comprising 2630 genes; Figure 4 and Table S9) were unique to *O. henryi* and were significantly enriched for pathways related to alanine, aspartate, and glutamate metabolism, diterpenoid biosynthesis, and photosynthesis (Figure 4B and Table S10). In this research, phylogenetic reconstruction indicated the closest relationship between *O. henryi* and *Lupinus albus*, with an estimated divergence time of ~53.82 million years ago (Figure 4C). Gene family expansion–contraction analysis revealed that, compared with the ancestral state, *O. henryi* exhibited significant expansion of 276 gene families, which were significantly enriched (q < 0.05) in pathways associated with protein export, RNA polymerase activity, and photosynthesis (Figure 4D and Table S11). In contrast, only nine gene families (containing 12 genes) underwent contraction, including genes regulating plant height, such as the ATP-dependent DNA helicase PIF1 (gene ID: Omi05G027420.1; Table S12). Positive selection analysis identified 122 genes (*p* < 0.05) enriched for base excision repair, spliceosome, and endoplasmic reticulum protein-processing pathways (Figure 4E and Table S13), highlighting adaptive evolution in core cellular maintenance pathways that are potentially critical for stress resilience.

### 2.4. Synteny

Genomic synteny analysis between *O. henryi* and representative Fabaceae species (Figure S4) identified 22, 20, and 20 syntenic blocks with *Cajanus cajan* (pigeon pea), *Medicago truncatula* (barrel medic), and *Glycine max* (soybean), respectively. These blocks corresponded to those of 33,380 (49.67%), 38,875 (46.58%), and 56,407 (59.36%) syntenic gene pairs (Table S14). These model legumes were prioritized over Lupinus albus for collinearity comparison because of their superior chromosome-level annotation quality, which provides a more rigorous benchmark for structural validation. This extensive collinearity not only confirms the structural integrity of the *O. henryi* assembly, a prerequisite for reliable conservation genomics, but also identifies highly conserved genomic frameworks essential for functional inference. The highest degree of synteny was observed between *O. henryi* and *G. max*, congruent with their phylogenetic proximity.

**Figure 5:**
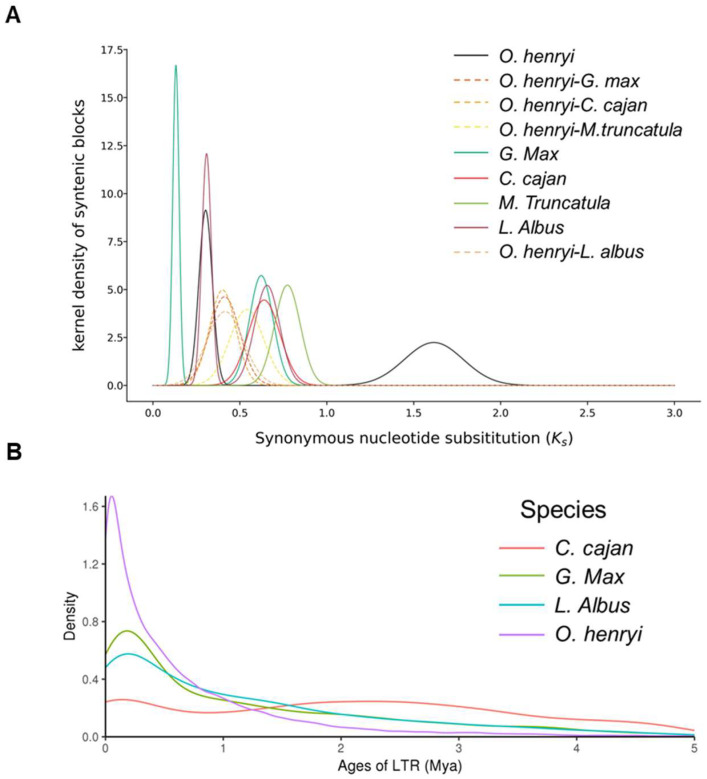
WGD (**A**) and LTR insertion time analysis (**B**).

WGD analysis identified two independent duplication events in *O. henryi* (*Ks* peaks at 0.47 and 2.26; Figure 5A), corroborated by 4DTv distribution data (Figure S5). Analysis of long terminal repeat (LTR) insertion times indicated a burst of activity within the last million years (<1 Mya; Figure 5B), with the expansion of Copia elements (~0.06 Mya) postdating that of Gypsy elements (Figure S6). This recent LTR-RT flux potentially reflects adaptive responses to Late Pleistocene climatic shifts. Such genomic plasticity, juxtaposed with the observed structural stability, provides a crucial genetic framework for evaluating the species’ evolutionary potential and formulating precision conservation strategies.

## 3. Discussion

### 3.1. Genome Resource Significance

Compared with that of earlier studies (Tables S15–S17), we successfully constructed a high-fidelity, chromosome-level reference genome (2.64 Gb; Contig N50 = 39.17 Mb; Scaffold N50 = 338.40 Mb; Hi-C anchoring rate = 97.8%) for the rare and endangered *O. henryi* [8,9,10]. Generating such a high-quality resource is a fundamental prerequisite for in-depth exploration of plant evolutionary history, environmental adaptive mechanisms, and the genetic basis of key traits in endangered timber species [11,12]. To effectively overcome the assembly challenges posed by the high heterozygosity (1.37%) and an exceptionally high repetitive sequence content (83.89%) of the *O. henryi* genome, we employed high-depth PacBio HiFi sequencing combined with Hi-C technology, balanced read length, and high accuracy. Compared with the recently published *O. henryi* genome [10], our assembly provides a more comprehensive functional landscape, particularly with respect to protein-coding gene completeness (99.32% vs. 88.42%; Tables S17–S19). These improvements in annotation depth address previous gaps in the species’ genomic repertoire.

A total of 38,753 protein-coding genes were annotated with high confidence. Compared with earlier assemblies of *Ormosia* species (e.g., *O. purpureiflora*, *O. emarginata*, *O. semicastrata,* and *O. boluoensis*) (Tables S15 and S16), this assembly demonstrates substantial refinements in gene model accuracy and functional coverage [8,9]. The integration of high-depth Hi-C data (165.10×) further ensures the structural integrity of the scaffolds, providing a validated and reliable genomic foundation. This work establishes a definitive reference for subsequent population genomics by resolving the complex repeat landscape and providing a near-complete gene set, enabling the identification of deleterious mutations and the assessment of genetic diversity, crucial steps for formulating effective conservation and restoration programs for *O. henryi*.

### 3.2. Evolutionary History and Its Implications for Conservation Genomics

While genome assembly provides fundamental genetic information, its utility for understanding the complex drivers of species evolution or temporal changes in gene function remains a subject of ongoing investigation [13]. For *O. henryi*, recorded data indicate a sharp decline in population size and significant habitat loss in relatively recent history, predominantly associated with intense external pressures. These include overexploitation driven by the high economic value of timber and medicinal products, as well as habitat fragmentation [14]. Given that the timescales of these disturbances (decades to centuries) are vastly shorter than the evolutionary timescales required for adaptive molecular mechanisms to arise (millions of years), it is reasonable to hypothesize that the current status of *O. henryi* reflects a potential mismatch between its long-term evolutionary pace and rapid environmental shifts.

Our comparative genomic analysis shows how *O. henryi* has historically navigated environmental complexity. Traditional views often attribute the species’ decline to inherent biological constraints, such as slow growth and low seed set [5,15]. However, the species demonstrates successful regeneration and persistence capacity in the absence of intense anthropogenic pressure, as evidenced by reports from specific geographic areas (e.g., *O. henryi* is a co-dominant or dominant canopy tree in certain areas) [16]. Furthermore, some populations exhibit successful seedling recruitment [17,18]. Moreover, *O. henryi* is one of the most adaptable plants distributed in southern China, which implies that the species has maintained a degree of evolutionary resilience over geological time [19]. The slow life-history strategy of *O. henryi* may have been an evolutionarily stable adaptation under historical natural disturbance regimes, though it appears increasingly vulnerable under current levels of exploitation [20].

Based on evidence from the existing in-depth comparative genomic analysis (WGD, gene family expansion, historical positive selection, and functional enrichment), it is clear that the tree has accumulated substantial genetic and functional diversity at the genomic level. Molecular evolutionary evidence indicates that *O. henryi* experienced two ancient WGD events, occurring ~58 Mya and earlier. The latter corresponds to the core-eudicot shared WGD, whereas the former aligns with the WGD event shared by the subfamily Papilionoideae [21]. Gene family evolution analysis has revealed significant gene family expansion (276 families) throughout the evolutionary history of *O. henryi*, with minimal contraction (only 9 families). These significantly expanded gene families are functionally enriched for pathways critical to environmental adaptation, including protein export, RNA polymerase activity, photosynthesis, phenylpropanoid biosynthesis, and the metabolism of alanine, aspartate, and glutamate. These expansions could enhance primary metabolism and provide precursors for secondary metabolites [22,23]. Critically, these expanded gene families and conserved genomic regions provide a valuable genomic reservoir for developing high-resolution molecular markers (e.g., SNPs) to monitor population health. Furthermore, the identification of 122 genes under positive selection, particularly those involved in DNA repair and proteostasis [24], indicates that *O. henryi* has historically evolved mechanisms to preserve genomic stability. Such markers will be essential for identifying stress-tolerant germplasm and guiding precision conservation efforts.

In conclusion, the genomic signatures of *O. henryi* point toward a history of successful adaptation to past environmental challenges. The current endangered status may be better understood as a consequence of recent external shocks that have overridden this established genetic framework. We can move toward a more proactive and evidence-based management strategy for this rare species by leveraging these genomic insights to develop robust conservation data.

### 3.3. Implications for Conservation and Sustainable Utilization

The high-quality genomic resource developed in this study bridges the gap between theoretical evolutionary insights and practical applications, thereby enabling the sustainable utilization of *O. henryi*. This resource not only facilitates the design of precision conservation strategies but also offers transformative opportunities for industrial applications, including high-value-added wood processing, the development of unique medicinal resources, ecological restoration, and landscape horticulture [25,26]. However, translating this genomic potential into tangible outcomes will require concerted efforts in conservation management, overcoming propagation bottlenecks, and establishing responsible harvesting and cultivation frameworks. Building upon the evolutionary signatures and candidate loci identified in the previous section, future research should focus on the following interconnected directions:

(1) Development of high-resolution molecular tools for precision conservation: Leveraging the 276 expanded gene families and 122 positively selected genes identified here, highly polymorphic molecular markers (e.g., SNPs and SSRs) should be immediately developed. These tools are essential for systematically assessing the genetic diversity and fine-scale population structure of extant populations, thereby guiding the formulation of evidence-based conservation units and the establishment of a core germplasm repository [27,28]. Concurrently, integrating population genomics with environmental variables will enable the identification of adaptive genetic loci, thereby facilitating the selection of stress-resistant lines (e.g., tolerance to drought, cold, or pests) to enhance the stability of restored populations [29,30].

(2) Elucidation of the genetic basis of key economic and adaptive traits: The metabolic robustness suggested by our genomic analysis, particularly within the phenylpropanoid biosynthesis and primary metabolism pathways, requires in-depth investigation. By integrating multi-omics technologies such as transcriptomics and metabolomics, researchers can identify core regulatory genes and key allelic variants. For instance, the annotated ATP-dependent DNA helicase *PIF1*, a known growth regulator in other species [31], warrants further functional validation in *O. henryi*. Coupling these insights with efficient somatic embryogenesis-based rapid propagation will facilitate molecular marker-assisted breeding [3], thereby enabling the development of new varieties with accelerated growth cycles and enhanced biomass accumulation.

(3) Implementation of genome-wide selection models: Large-scale breeding populations should be established to accelerate the genetic improvement of complex traits, such as wood quality and multi-stress resistance. Utilizing the genome-wide markers derived from this assembly will enable the construction of genomic selection models, significantly reducing the breeding cycle for this long-lived woody species.

(4) Expansion to genus-wide comparative research: The methodologies and genomic resources generated for *O. henryi* should be extended to other economically or ecologically significant species within the genus *Ormosia*. This broader comparative framework will promote systematic research into the genus’s evolutionary history and support the sustainable development of these rare forest resources across their entire distribution range.

## 4. Materials and Methods

### 4.1. Plant Material

Healthy *O. henryi* seedlings (n = 3; three-year-old) were sampled from the nursery of the Hunan Botanical Garden (28°20′ N, 113° E; altitude: 70–85 m). Root, stem, and leaf tissues were collected separately from each plant. Thereafter, the samples were flash-frozen in liquid nitrogen and stored at −80 °C.

### 4.2. Genomic Sequencing

A multi-platform sequencing strategy was employed for genome sequencing. Initially, high-quality genomic DNA was extracted from young leaves using a plant DNA extraction kit (TIANGEN, Beijing, China). This DNA was used to construct an Illumina library with an insert size of ~350 bp, which was sequenced using the DNBSEQ-G400 platform (MGI Tech Co., Ltd., Shenzhen, China). Raw data were subjected to quality control and filtering using SOAPnuke v1.5.6.

Subsequently, HiFi sequencing was performed. DNA was sheared using a Megaruptor (Diagenode, Liège, Belgium), and fragments of 13–16 kb were size-selected using a Sage ELF system (Sage Science, Beverly, MA, USA). A 20 kb SMRTbell library was constructed and sequenced in Circular Consensus Sequencing (CCS) mode on the PacBio Sequel II platform (Pacific Biosciences, Menlo Park, CA, USA). CCS v5.0.0 processing yielded 96.52 Gb of HiFi clean data.

For chromosomal conformation capture (Hi-C) analysis, fresh leaf tissue was cross-linked with formaldehyde. Cells were lysed, and genomic DNA was digested with the restriction enzyme MboI (New England Biolabs, Ipswich, MA, USA). Following end repair, biotin labeling was introduced, and proximity ligation was performed using DNA ligase. After protease digestion to reverse cross-links, DNA was purified, sheared to 300–700 bp, and interacting fragments were specifically captured using streptavidin beads to construct the Hi-C library. Library quality was assessed using Qubit 2.0 (Thermo Fisher Scientific, Waltham, MA, USA) and Agilent 2100 (Agilent Technologies, Santa Clara, CA, USA), with effective concentration validated by qPCR prior to sequencing on the DNBSEQ platform (MGI Tech Co., Ltd., Shenzhen, China) (insert size: 350 bp), generating ~434.2 Gb of valid data. Contigs were subsequently clustered, ordered, and anchored into chromosomal scaffolds using 3D-DNA v180114, followed by manual correction of scaffold order and orientation using Juicebox v2.1.0.

Additionally, total RNA was extracted from *O. henryi* root, stem, and leaf tissues. Following library construction and quality control (Qubit 2.0, Agilent 2100), RNA sequencing was performed using the DNBSEQ platform. Data quality was assessed using FastQC v0.11.8.

### 4.3. Genome Assembly and Assessment

Assembly commenced with a k-mer frequency analysis of Illumina sequencing data using Jellyfish v2.1.4, followed by estimation of genome size, heterozygosity, and repeat content using the Genomescope model. The PacBio HiFi reads were then assembled de novo using Hifiasm v0.16, and redundant sequences were subsequently removed using purge_dups. Finally, Hi-C sequencing data were used to anchor the assembled contigs to chromosomes, yielding a high-quality chromosome-level genome assembly for *O. henryi*.

Assembly quality was assessed through three complementary approaches: (1) genome completeness was evaluated using BUSCO v5.2.2 with the embryophyta_odb10 dataset; (2) base-level accuracy was assessed by mapping Illumina short reads to the assembly using BWA v0.7.17; and (3) spatial organization validation was achieved by generating a normalized heatmap from Hi-C interaction data, where the characteristic diagonal pattern of intra-chromosomal interactions confirmed the structural integrity of the pseudochromosomes.

### 4.4. Gene Prediction and Annotation

The assembled genome underwent comprehensive annotation, encompassing repetitive sequences, protein-coding genes, functional annotation against multiple databases (NR, eggNOG, GO, KEGG, TrEMBL, SwissProt, KOG, Pfam, and InterPro), pseudogene identification, and non-coding RNA (ncRNA) annotation.

Repetitive sequence annotation employed a hierarchical strategy: De novo prediction was conducted using RepeatModeler v2.0.1, integrating RECON v1.0.8 and RepeatScout v1.0.6. LTR retrotransposon structures were identified explicitly with LTR_retriever v2.9.0, and the combined custom repeat library was used to mask repetitive regions using RepeatMasker v4.1.2. Tandem repeats were detected jointly using MISA v2.1 and TRF v4.0.9.

The protein-coding gene prediction integrated evidence from three sources: (1) ab initio prediction using Augustus v3.1.0 and SNAP; (2) homology-based prediction with GeMoMa v1.7 against protein sequences from nine legume species (*Arachis hypogaea*, *Cajanus cajan*, *Glycine max*, *Lupinus albus*, *L. angustifolius*, *Lotus japonicus*, *Oxytropis ochrocephala*, *Phaseolus vulgaris*, and *Trifolium pratense*); and (3) transcriptome evidence derived by mapping RNA-seq data to the genome using Hisat2 v2.1.0 and StringTie v2.1.4, with de novo transcript assembly provided by Trinity v2.8.5. Predictions from these methods were integrated and optimized using EVM v1.1.1, and UTR annotations, along with alternative splicing isoforms, were further refined using PASA v2.3.3.

Non-coding RNA annotation involved tRNA identification using tRNAscan-SE v1.3.1, rRNA prediction with Barrnap v0.9, and identification of other RNAs (miRNA, snRNA, and snoRNA) via alignment against the Rfam database.

Pseudogenes were predicted by initially screening for homologous sequences with GenBlastA v1.0.4, followed by detection of frameshift mutations and premature stop codons with GeneWise v2.4.1.

Functional annotation was performed by conducting BLAST v2.2.23 searches against eight major databases (NR, SwissProt, TrEMBL, KOG, Pfam, InterPro, GO, and KEGG). The completeness of the final annotated gene set was assessed using BUSCO.

### 4.5. Gene Family Clustering

Orthologous clustering analysis of the complete protein sequences from *Ormosia henryi* and nine other legume species (Table S1) was performed using OrthoFinder v2.4 (DIAMOND alignment; e-value threshold: 1 × 10^−5^). Following gene family classification, functional annotation was performed using the PANTHER v15 database. Gene Ontology (GO) functional enrichment and KEGG pathway enrichment analyses (significance threshold q-value < 0.05) were performed explicitly on *O. henryi*-specific gene families using clusterProfiler v3.4.4.

### 4.6. Phylogeny

A species phylogenetic tree was reconstructed based on 444 single-copy orthologous genes. Multiple sequence alignment was first performed using MAFFT v7.205 (parameters: localpair maxiterate 1000). Low-confidence alignment regions were filtered using Gblocks v0.91b (parameters: −b5 = h) to enhance data quality. Subsequently, the maximum-likelihood tree was constructed using IQ-TREE v1.6.11, in which the ModelFinder module identified the JTT + F + I + G4 model as the optimal substitution model. Node support was assessed using 1000 bootstrap replicates. Divergence times were estimated using MCMCTree within the PAML package, with *Oryza sativa* as the outgroup to calibrate the evolutionary timescale.

### 4.7. Gene Family Expansion/Contraction

Gene family dynamics were analyzed using CAFE v4.2. Inputting the phylogenetic tree topology and gene family clustering results, the software inferred gene family counts at ancestral nodes via birth–death models and detected significant expansion/contraction events along modern species branches (likelihood ratio test *p* < 0.01). KEGG pathway and GO functional enrichment analyses were separately performed on expanded and contracted gene families to elucidate functional evolutionary trends.

### 4.8. Positive Selection

Positive selection signals were detected using the branch-site model in CodeML (PAML v4.9). Single-copy gene families shared between *O. henryi* and closely related species (*C. cajan*, *G. max*, etc.) were screened. Protein alignments were converted to codon-based alignments using PAL2NAL. Likelihood ratio tests (LRTs) were conducted by comparing the fit of Model A (allowing ω > 1 on foreground branches, with *O. henryi* defined as foreground branches) against that of the null model (ω ≤ 1 globally). Genes showing significant LRT differences (*p* < 0.05) were further analyzed using the Bayes Empirical Bayes (BEB) method to identify positively selected sites (a posterior probability of >0.95 was considered significant).

### 4.9. WGD Analysis

Paleopolyploidy events were investigated using synteny analysis and molecular clock modeling. Syntenic blocks between *O. henryi* and *C. cajan*, *Medicago truncatula*, and *G. max* were identified using MCScanX (parameter: −m 25). Homologous gene pairs within these blocks were extracted, and their synonymous substitution rates (Ks) were calculated using PAML. Ks distribution histograms were plotted to detect peaks indicative of WGD event timing. Additionally, the rate of transversions at four-fold degenerate sites (4DTv) was calculated, and its bimodal distribution was used to validate the number and timing of paleopolyploidization events.

### 4.10. LTR Insertion Time

Candidate long terminal repeat retrotransposon (LTR-RT) sequences were identified using LTR_FINDER v1.07 and LTRharvest v1.5.9 in parallel, with results integrated and filtered by LTR_retriever v2.8. The 5′ and 3′ LTR sequences of each element were extracted to estimate the insertion time. These sequences were aligned using MAFFT (parameters: localpair maxiterate 1000). The Kimura 2-parameter genetic distance (K) was calculated using the EMBOSS v6.6.0 distmat program. The insertion time (T) was calculated using the formula T = K/(2 × r), where the molecular clock rate (r) was set to 7 × 10^−9^ substitutions per site per year.

## 5. Conclusions

In this study, a high-quality, chromosome-level reference genome for *O. henryi* was successfully constructed, effectively overcoming the assembly challenges posed by high heterozygosity (1.37%) and repetitive sequence content (83.89%). Systematic genomic analyses, including the identification of 2 ancient WGD events, 276 expanded gene families, and 122 genes under positive selection, suggest that the species has maintained substantial evolutionary resilience and adaptive potential throughout its history. While the rapid decline in extant populations underscores the overwhelming impact of external anthropogenic pressures, the demonstrated genomic stability and metabolic robustness offer a vital foundation for recovery. Consequently, this genomic resource provides a critical framework for screening individuals with high adaptive fitness to facilitate both population restoration and industrial utilization. This study leverages these molecular insights to balance resource protection with sustainable application, promoting a synergistic approach to the conservation and development of *O. henryi* and the broader genus *Ormosia*.

## Figures and Tables

**Table 1:** Summary of *Ormosia henryi* genome assembly.

Statistic	Scaffold	Contig
Total number	361	537
Total length of (bp)	2,639,023,864	2,638,935,864
Gap number (bp)	586,000	0
N50 length (bp)	338,397,820	39,169,180
N90 length (bp)	240,162,212	9,385,987
Maximum length (bp)	380,859,801	148,418,790
Minimum length (bp)	240,162,212	9,385,987

**Table 2:** Genome assembly evaluation using BUSCO.

Type	Contig Level		Chromosome Level	
	Number	Percentage (%)	Number	Percentage (%)
Complete BUSCOs (C)	1586	98.3	1587	98.3
Complete and single-copy BUSCOs (S)	1464	90.7	1464	90.7
Complete and duplicated BUSCOs (D)	122	7.6	123	7.6
Fragmented BUSCOs (F)	11	0.7	10	0.6
Missing BUSCOs (M)	17	1.0	17	1.1
Total BUSCO groups searched	1614	-	1614	-

**Table 3:** Statistical results of gene functional annotations.

Database	Number	Percentage (%)
GO_Annotation	31,181	79.92
KEGG_Annotation	30,352	77.79
KOG_Annotation	22,984	58.91
Pfam_Annotation	32,964	84.49
Swissprot_Annotation	28,316	72.57
TrEMBL_Annotation	38,735	99.28
eggNOG_Annotation	33,323	85.41
nr_Annotation	38,532	98.76
All_Annotated	38,753	99.32

## Data Availability

The whole-genome sequence data of *Ormosia henryi* reported in this paper have been deposited in the Genome Warehouse (GWH) of the National Genomics Data Center, Beijing Institute of Genomics, Chinese Academy of Sciences/China National Center for Bioinformation, under accession number GWHFICR00000000.1, publicly accessible at https://ngdc.cncb.ac.cn/gwh (accessed on 4 January 2026). In addition, this Whole Genome Shotgun project has been deposited at DDBJ/ENA/GenBank under the accession JBTHJL000000000; the version described in this paper is JBTHJL010000000. The corresponding NCBI records are linked to BioProject PRJNA1395312 and BioSample SAMN54355153 (Submission ID: SUB15898749). These data are scheduled for release on 31 January 2026, or upon publication, whichever occurs first. To facilitate reuse, the genome annotation files are available on Zenodo at https://doi.org/10.5281/zenodo.18093271.

## Supplementary Materials

The following supporting information can be downloaded at: https://www.mdpi.com/article/10.3390/plants15020180/s1, Figure S1: 21-mer frequency distribution curve of the *Ormosia henryi* genome; Figure S2: GO annotation and functional classification of the *Ormosia henryi* genome; Figure S3: eggNOG/KEGG function classification of consensus sequences in the *Ormosia henryi* genome; Figure S4: Collinearity analysis between *Ormosia henryi* and representative Fabaceae species (*C. cajan*, *M. truncatula*, and *G. max*); Figure S5: 4DTv distribution map for *Ormosia henryi* and other representative legume species; Figure S6: The insertion time distribution of Copia, Gypsy, and unknown LTR-RT elements in the *Ormosia henryi* genome; Table S1: Genomic information and data sources of closely related species used in the comparative analysis; Table S2: Summary of *Ormosia henryi* genome sequencing data from PacBio HiFi and Hi-C platforms; Table S3: Statistics of the genomic characteristics of *Ormosia henryi* obtained by 21-mer genome survey analysis; Table S4: Detailed statistics of repeat sequences, including interspersed and tandem repeats, in the *Ormosia henryi* genome; Table S5: Statistical results of non-coding RNA (rRNA, tRNA, miRNA, snRNA, and snoRNA) annotation; Table S6: Prediction results and length statistics of pseudogenes in the *Ormosia henryi* genome; Table S7: Statistics of protein-coding gene prediction results using Ab initio, homology-based, and RNA-seq integration methods; Table S8: BUSCO assessment of the completeness and reliability of genomic function predictions; Table S9: Statistical information sheet of gene family clustering across ten species; Table S10: Functional annotation and enrichment analysis of unique gene families in *Ormosia henryi*; Table S11: Comprehensive functional annotation of significantly expanded gene families in *Ormosia henryi* based on multiple databases; Table S12: Detailed information and functional annotation of contracted gene families in *Ormosia henryi*; Table S13: Functional annotation and enrichment analysis of genes subject to positive selection (PSGs) in *Ormosia henryi*; Table S14: Statistics of syntenic blocks and collinear gene analysis between *O. henryi* and related species; Table S15: Data comparison between our results and Liu et al. (2023) [9] results; Table S16: Data comparison between our results and Wang et al. (2025) [8] results; Table S17: Data comparison between our results and Zhou et al. (2025) [10] results; Table S18: Data comparison between our results and Zhu et al. (2025) [7] results; Table S19: Data comparison between our results and other *Ormosia* species.
