# Supplementary material for: Chromosome-Level Genome Assembly of Ormosia henryi Provides Insights into Evolutionary Resilience and Precision Conservation
Source: Plants (Basel). 2026 Jan 7;15(2):180. doi: 10.3390/plants15020180 (PMC12845328; doi:10.3390/plants15020180)
Supplement: Supplementary file 1 [file plants-15-00180-s001.zip › Table S1-8 & Fig 1-6.pdf]

Table S1 Genomic information and data sources of closely related species used in the comparative analysis

Name	Species	Download
Tprat	*T. pratense*	https://ftp.ensemblgenomes.ebi.ac.uk/pub/plants/release-56/fasta/trifolium_pratense/dna/
Lalbu	*L. albus*	https://ftp.ensemblgenomes.ebi.ac.uk/pub/plants/release-58/fasta/lupinus_angustifolius/dna/
Ohenr	*O. henryi*	GWHFICR00000000.1
Gmax	*G. max*	https://ngdc.cncb.ac.cn/gwh/Assembly/66216/show
Osati	*O. sativa*	http://www.ricesuperpir.com/web/download
Adura	*A. duranensis*	https://ftp.ncbi.nlm.nih.gov/genomes/all/GCF/000/817/695/GCF_000817695.2_Aradu1.1/
Mtrun	*M. truncatula*	https://ftp.ncbi.nlm.nih.gov/genomes/all/GCA/003/473/485/GCA_003473485.2_MtrunA17r5.0-ANR/
Ljapo	*L. japonicus*	https://phytozome-next.jgi.doe.gov/info/Ljaponicus_Lj1_0v1
Ccaja	*C. cajan*	https://ftp.ncbi.nlm.nih.gov/genomes/all/GCF/000/340/665/GCF_000340665.2_C.cajan_V1.1/
Pvulg	*P. vulgaris*	https://phytozome.jgi.doe.gov/pz/portal.html#!bulk?org=Org_Pvulgaris

Table S2 Summary of *Ormosia henryi* genome sequencing data from PacBio HiFi and Hi-C platforms

Libraries	Read_base	Read_Number	Depth (×)
PacBio HiFi reads	96,521,315,992	6,034,056	36.7
Hi-C Illumina reads	434,211,000,000	436,140,000,000	165.1

Table S3 Statistics of the genomic characteristics of *Ormosia henryi* obtained by 21-mer genome survey analysis

K	K-mer Number	Genome Size(bp)	Repeat (%)	Heterozygous Ratio (%)	Used Bases(bp)	Sequence Depth(X)
21	52348068609	2625603176	77.46	1.37	120.845286	46.03

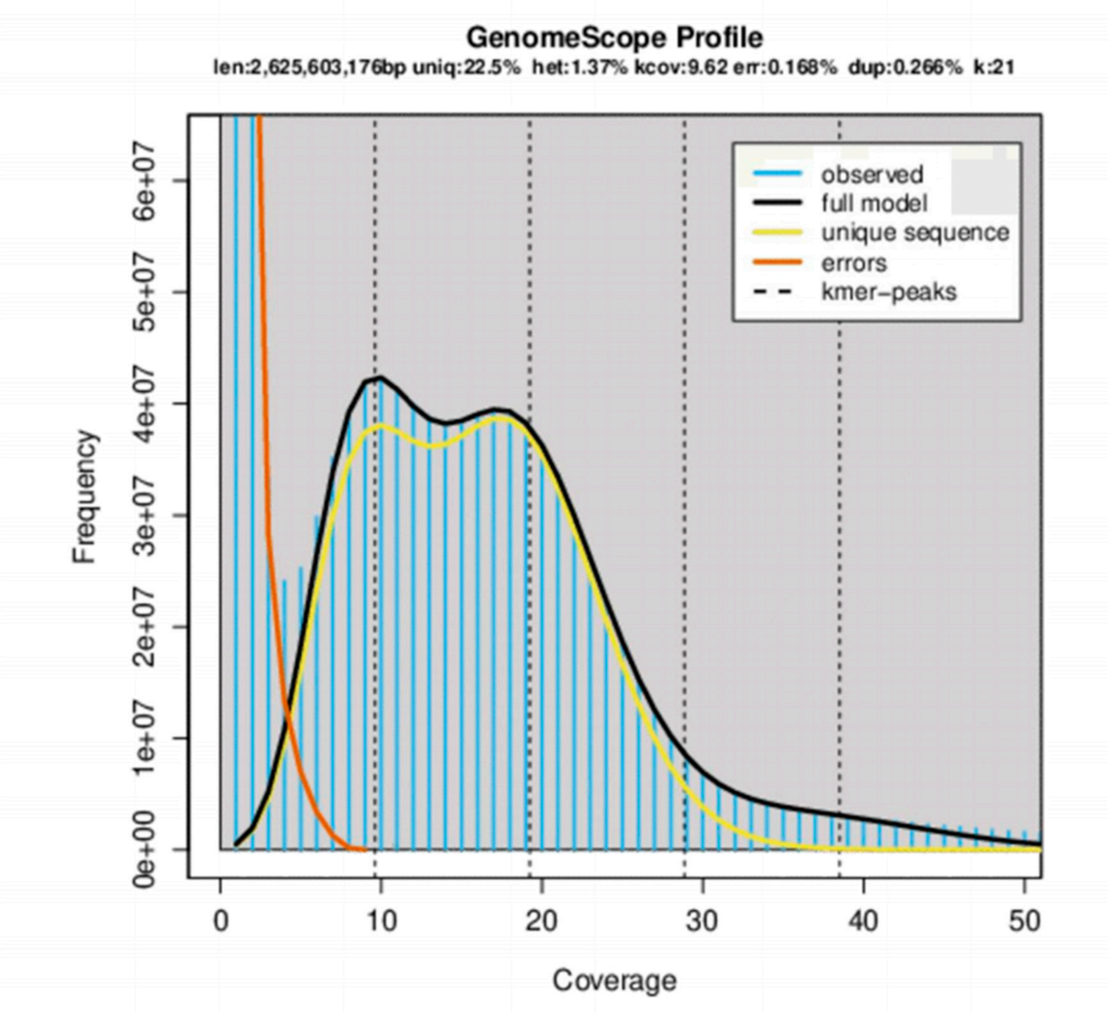

Figure S1 21-mer frequency distribution curve of the *Ormosia henryi* genome

Table S4 Detailed statistics of repeat sequences, including interspersed and tandem repeats, in the *Ormosia henryi* genome

Type (interspersed repeats)	Number	Length	Rate(%)
ClassI:Retroelement	1388114	1.967E+09	74.55
ClassI/DIRS	2	95	0
ClassI/LINE	28963	10328491	0.39
ClassI/LTR/Copia	182994	174462218	6.61
ClassI/LTR/ERV	2928	222635	0.01
ClassI/LTR/Gypsy	631036	1.354E+09	51.29
ClassI/LTR/Ngaro	340	25044	0
ClassI/LTR/Pao	182	59066	0

ClassI/LTR/Unknown	540059	428316882	16.23
ClassI/SINE	1610	335655	0.01
ClassII:DNA transposon	231943	102938383	3.9
ClassII/Academ	2	101	0
ClassII/CACTA	5686	3058717	0.12
ClassII/Crypton	49	1886	0
ClassII/Dada	427	19948	0
ClassII/Ginger	108	4784	0
ClassII/Helitron	143580	69062258	2.62
ClassII/IS3EU	191	10773	0
ClassII/Kolobok	710	60410	0
ClassII/Maverick	157	10908	0
ClassII/Merlin	289	13687	0
ClassII/Mutator	1866	1391467	0.05
ClassII/P	279	22736	0
ClassII/PIF-Harbinger	1079	62506	0
ClassII/PiggyBac	175	7427	0
ClassII/Tc1-Mariner	247	14280	0
ClassII/Unknown	74062	28938095	1.1
ClassII/Zisupton	114	5498	0
ClassII/hAT	2922	252902	0.01
Unknown	27	1580	0
Total	1620084	2.07E+09	78.45

Type (tandem repeats)	Number	Length	Rate(%)
Microsatellite (1-9 bp units)	1021584	22618692	0.86
Minisatellite (10-99 bp units)	1203698	91182220	3.46
Satellite (>=100 bp units)	64483	29760847	1.13
Total	2289765	143561759	5.44

Table S5 Statistical results of non-coding RNA (rRNA, tRNA, miRNA, snRNA, and snoRNA) annotation

rRNA_num	tRNA_num	miRNA_num	snRNA_num	snoRNA_num
9,174	2,068	74	201	2,619

Table S6 Prediction results and length statistics of pseudogenes in the *Ormosia henryi* genome

Pseudogene	Stat
Total_Number	776
Total_len	5,649,925
Average_Len	7280.83

Table S7 Statistics of protein-coding gene prediction results using *Ab initio*, homology-based, and RNA-seq integration methods

Method	Software	Species	Gene number
Ab initio	Augustus	-	58,048
	SNAP	-	42,138
Homology-based	GeMoMa	A. hypogaea	44,006
		C. cajan	36,851
		G. max	46,259
		L. albus	49,152
		L. angustifolius	30,333
		L. japonicus	33,523
		O. ochrocephala	32,467
		P. vulgaris	31,904
		T. pratense	36,107
RNAseq	GeneMarkS-T	-	23,593
	PASA	-	24,639
Integration	EVM	-	39,017

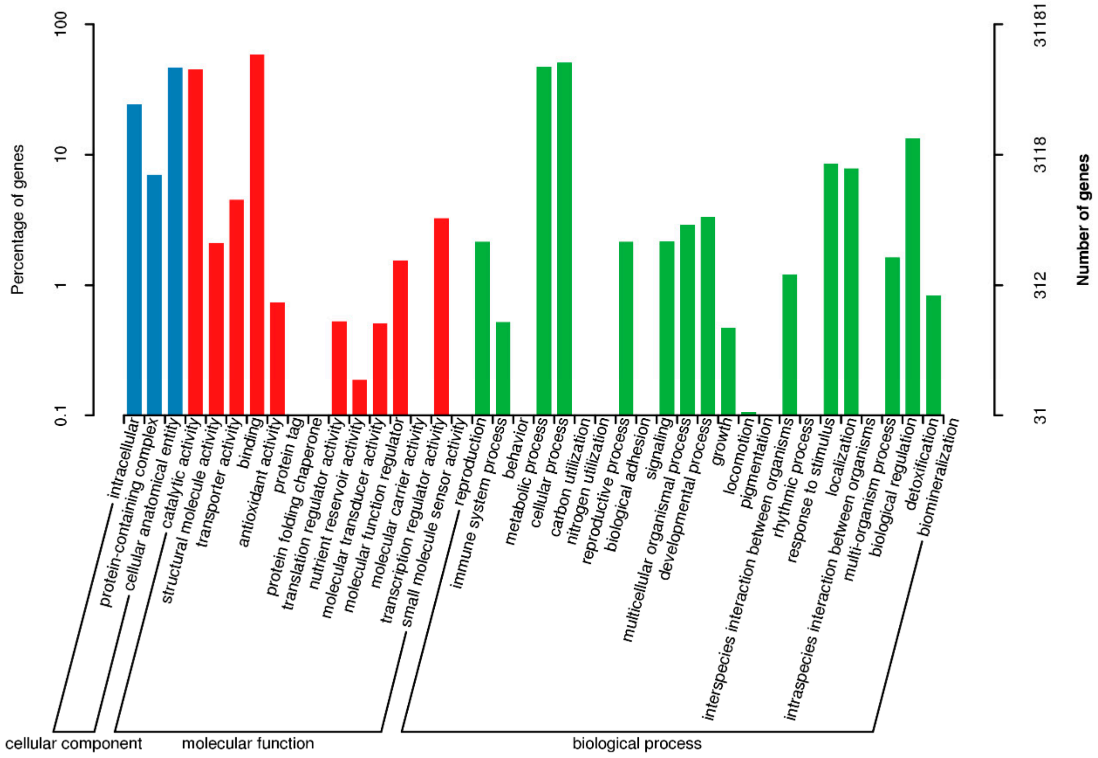

Figure S2 GO annotation and functional classification of the *Ormosia henryi* genome

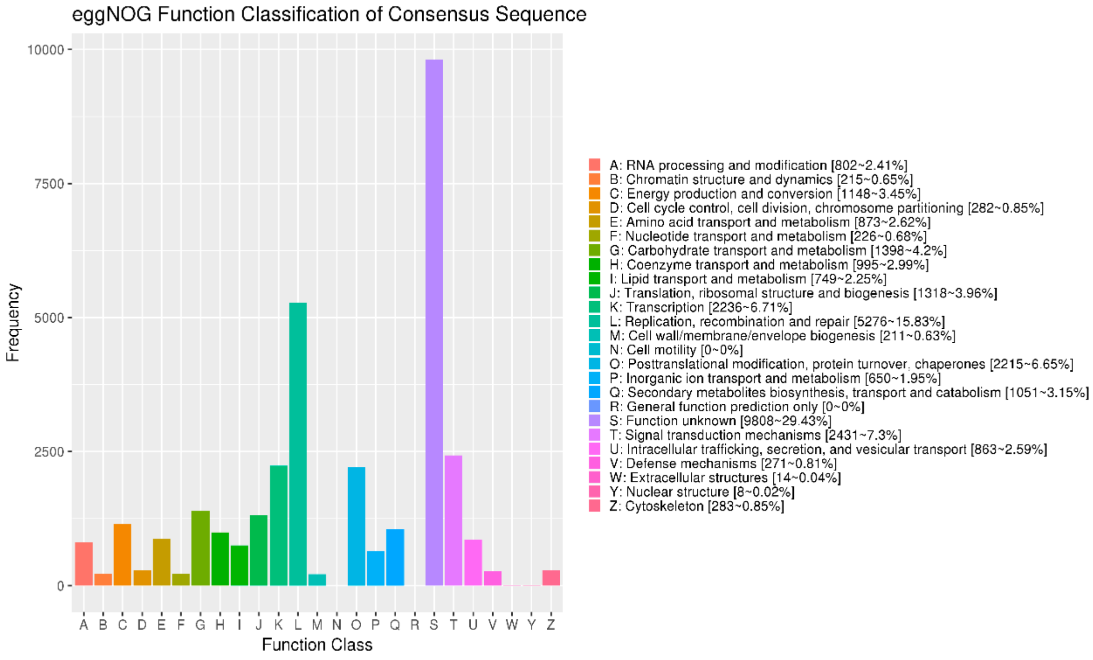

Figure S3 eggNOG/KEGG function classification of consensus sequences in the *Ormosia henryi* genome

Table S8 BUSCO assessment of the completeness and reliability of genomic function predictions

Type	Number (Percentage %)
Complete BUSCOs(C)	1591 (98.57%)
Complete and single-copy BUSCOs(S)	1472 (91.20%)
Complete and duplicated BUSCOs(D)	119 (7.37%)
Fragmented BUSCOs(F)	5 (0.31%)
Missing BUSCOs(M)	18 (1.12%)
Total Lineage BUSCOs	1614

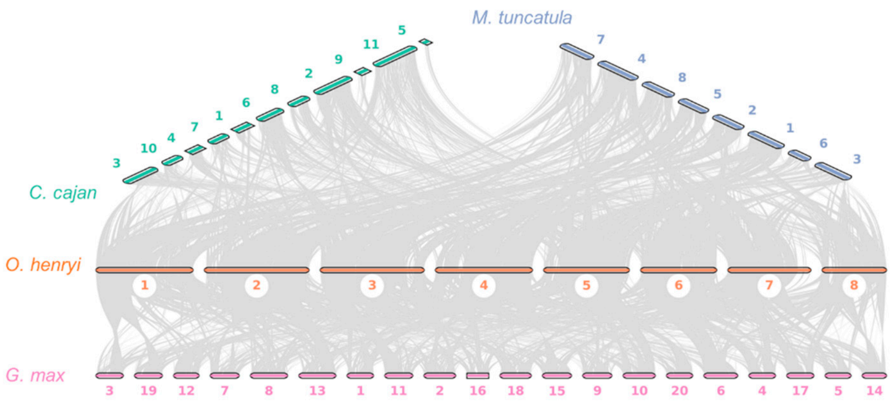

Figure S4 Collinearity analysis between *Ormosia henryi* and representative Fabaceae species (*C. cajan*, *M. truncatula*, and *G. max*)

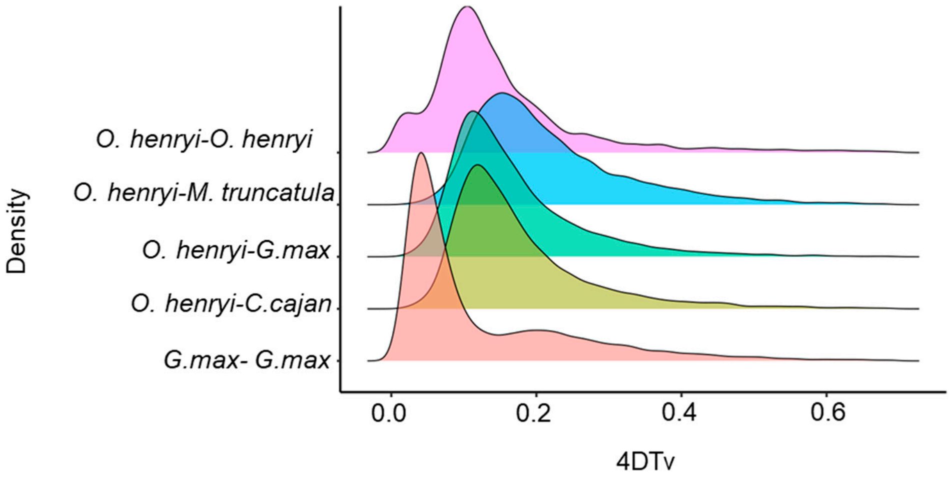

Figure S5 4DTv distribution map for *Ormosia henryi* and other representative legume species

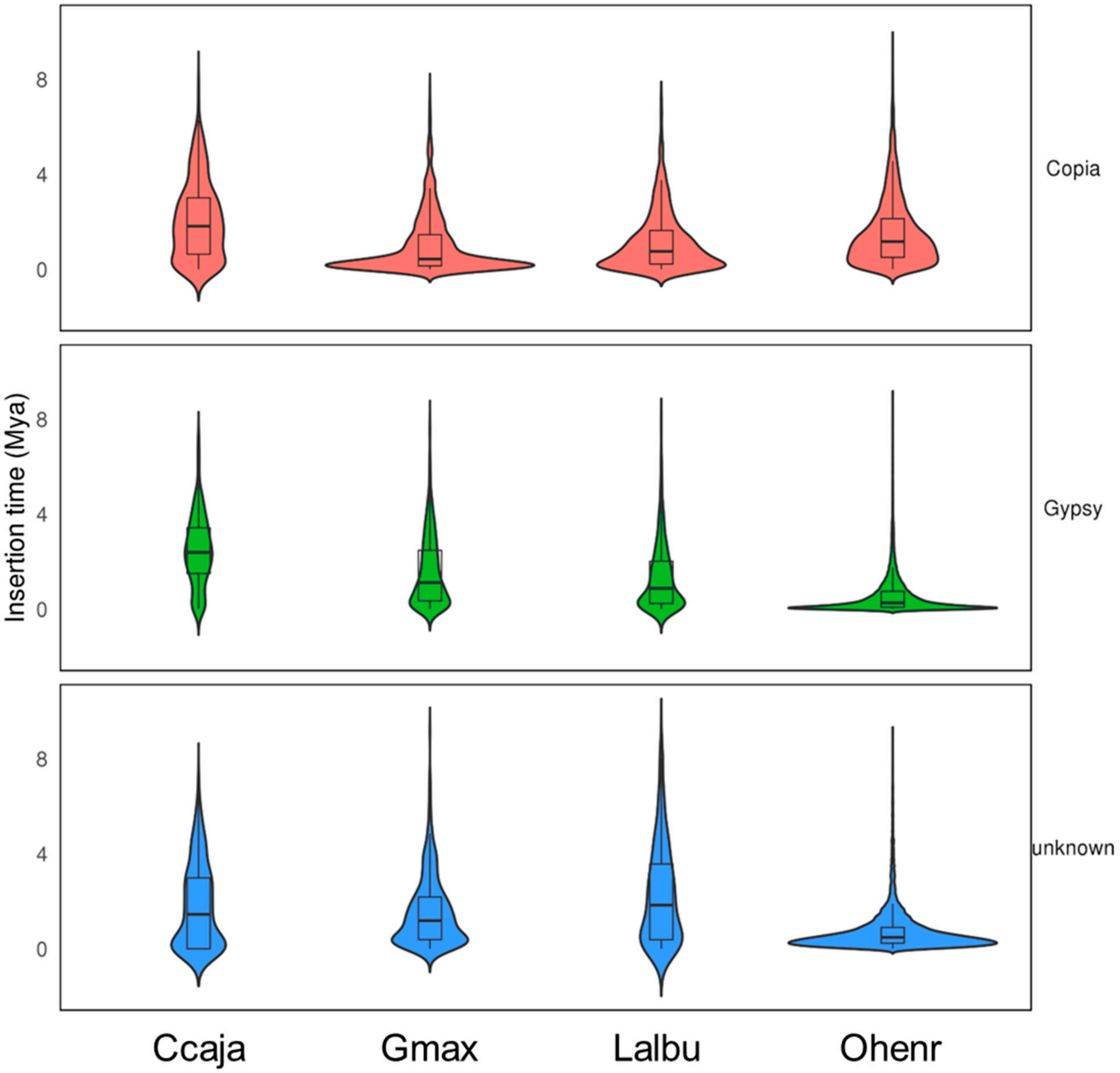

Figure S6 The insertion time distribution of Copia, Gypsy, and unknown LTR-RT elements in the *Ormosia henryi* genome
