# Supplementary material for: Chromosome-Level Genome Assembly of Ormosia henryi Provides Insights into Evolutionary Resilience and Precision Conservation
Source: Plants (Basel). 2026 Jan 7;15(2):180. doi: 10.3390/plants15020180 (PMC12845328; doi:10.3390/plants15020180)
Supplement: Supplementary file 1 [file plants-15-00180-s001.zip › Table S14.pdf]

**Table S14** Statistics of syntenic blocks and collinear gene analysis between *O. henryi* and related species

| | Ohenr_Ccaja | Ohenr_Mtrun | Ohenr_Gmax |
|---|---|---|---|
| Number of syntenic blocks | 22 | 20 | 20 |
| Number of collinear genes | 33380 | 38875 | 56407 |
| Percentage | 49.67 | 46.58 | 59.36 |
| Number of all genes | 67203 | 95024 | 95024 |
