# Supplementary material for: Chromosome-Level Genome Assembly of Ormosia henryi Provides Insights into Evolutionary Resilience and Precision Conservation
Source: Plants (Basel). 2026 Jan 7;15(2):180. doi: 10.3390/plants15020180 (PMC12845328; doi:10.3390/plants15020180)
Supplement: Supplementary file 1 [file plants-15-00180-s001.zip › Table S15-19.pdf]

Table S15 Data comparison between our results and Liu et al. (2023) results

Genomic Feature	*Ormosia henryi*	*O. emarginata*	*O. semicastrata*
Genome Size (Gb)	2.64	1.42	1.51
Contig N50 (Mb)	39.17	28.2	49.98
Scaffold N50 (Mb)	338.4	/	/
BUSCO Completeness (%)	98.3	97	98.3
Sequencing Platform	PacBio HiFi + Hi-C + Illumina	Oxford Nanopore + Illumina	
Repetitive Sequences (%)	83.89	74.12	75.24
Annotated Genes (%)	99.32	72.71	72.09

Reference:

Liu, P.-P., Yu, E.-P., Tan, Z.-J., Sun, H.-M., Zhu, W.-G., Wang, Z.-F., Cao, H.-L., 2023. Genome assemblies of two *Ormosia* species: gene duplication related to their evolutionary adaptation. Agronomy 13, 1757.http://doi.org/10.3390/agronomy13071757

Table S16 Data comparison between our results and Wang et al. (2025) results

Genomic Feature	*Ormosia henryi*	*O. purpureiflora*	*O. emarginata*	*O. semicastrata*
Genome size (Gb)	2.64	1.5	1.42	1.51
Contig N50 (Mb)	39.17	50.91	28.2	49.98
Scaffold N50 (Mb)	338.4	Chromosome-level assembly was performed but no N50 value was given		
BUSCO completeness (%)	98.3	98.3	97	98.4
Repetitive content (%)	83.89	76.3	74	75.2
Gene annotation rate (%)	99.32	70.81	76.43	72.43

Reference:

Wang, Z.-F., Yu, E.-P., Fu, L., Deng, H.-G., Zhu, W.-G., Xu, F.-X., Cao, H.-L., 2025. Chromosome-scale assemblies of three *Ormosia* species: repetitive sequences distribution and structural rearrangement. GigaScience 14, giaf047.https://doi.org/10.1093/gigascience/giaf047

Table S17 Data comparison between our results and Zhou et al. (2025) results

Genomic Feature	*Ormosia henryi* **(this paper)**	*O. henryi* **(Zhou's paper)**
Genome size (Gb)	2.64	2.69
Contig N50 (Mb)	39.17	37.59
Scaffold N50 (Mb)	338.40	354.08
Chromosome anchoring rate (%)	97.80	99.97
BUSCO completeness (%)	98.30 (Single-copy: 90.7%; Duplicated: 7.6%)	98.20 (Single-copy: 91.20%; Duplicated: 7.00%)
Repetitive content (%)	83.89	69.06
Sequencing depth (×)	HiFi: 36.70<br>Hi-C: 165.10	HiFi: 31.74<br>Hi-C: 50.92
Protein-coding genes	39017	42260
Functional annotation rate (%)	99.32 (38,753 genes)	88.42 (37,366 genes)
Non-coding RNA annotation	Novel miRNAs/snRNAs/snoRNAs (2,954 total)	Conventional types only (rRNAs/tRNAs)

Reference:

Zhou, C., Wen, Q., Zeng, D., Guo, C., Guo, Z., Liu, L., Ouyang, T., 2025. Chromosome-level genome assembly of the endangered tree species *Ormosia henryi* Prain. Scientific Data 12, 1065.https://doi.org/10.1038/s41597-025-05402-0

Table S18 Data comparison between our results and Zhu et al. (2025) results

Genomic Feature	*Ormosia henryi* **(this paper)**	*O. boluoensis***(Zhu's paper)**
Genome size (Gb)	2.64	1.64
Contig N50 (Mb)	39.17	15.65
Scaffold N50 (Mb)	338.40	201.05
Chromosome anchoring rate (%)	97.80	99.70
BUSCO completeness (%)	98.30 (Single-copy: 90.7%; Duplicated: 7.6%)	98.20
Repetitive content (%)	83.89	75.06
Sequencing depth (×)	HiFi: 36.70<br>Hi-C: 165.10	Short-read WGS: 78<br>Hi-C: 95
Protein-coding genes	39017	51,822
Functional annotation rate (%)	99.32 (38,753 genes)	72.45%(40,750 genes)
Non-coding RNA annotation	Novel miRNAs/snRNAs/snoRNAs (2,954 total)	No relevant report

Reference:

Zhu, JP., Wang, ZF., Cheng, F. et al. A chromosome-scale assembly of Ormosia boluoensis (Fabaceae). Sci Data 12, 1659 (2025). https://doi.org/10.1038/s41597-025-05953-2

Table S19 Data comparison between our results and other *Ormosia* species

Species	*O. purpureiflora*	*O. emarginata*	*O. semicastrata*	*O. boluoensis*	*Ormosia henryi (zhou)*	*Ormosia henryi(this paper)*
**Statistics and evaluations of genome assemblies for five *Ormosia* species**						
Genome size (Gb)	1.81	1.42	1.51	1.64	2.69	2.64
Contig N50 (Mb)	50.90	28.19	48.97	15.65	37.59	39.17
Scaffold N50 (Mb)	—	—	—	201.05	354.08	338.40
Gap number (bp)Scaffold/ Contig	645468(Scaffold)	142025(Scaffold)	453206(Scaffold)	—	—	586,000/0(Scaffold/Contig)
Average length	5,786,506.08 bp	15,787,973.39 bp	23,996,300.94 bp	9,102,694.42 bp	—	7,310,315 bp
Largest length	142,757,542 bp	84,853,091 bp	144,833,628 bpp	236629900 bp	—	380,859,801
Minimum length	34,487 bp	173,104 bp	128,272 bp	1000 bp	—	240,162,212
Chromosome anchoring rate (%)	99.96%	99.99%	99.96%	99.70%	97.80%	99.97%
GC content	35.06%	34.53%	34.63%	34.60%	35.68%	36.00%
Percentage of total repeat sequences	65.5%	62.4%	64.1%	75.06%	69.06%	83.89 %
Number of predicted	55,061	50,517	51,220	51,822	42,260	39,017

genes						
BUSCO completeness at genome level	98.3%	97%	98.4%	98.2%	—	—
BUSCO completeness at gene level	—	—	—	96.7%	98.20%	98.30%
Complete BUSCOs(C)	98.3%	97%	98.4%	98.20%	1585 (98.20%)	1587（98.3）
Complete and single-copy BUSCOs(S)	89.4%	89.4%	90.4%	90.2%	1472 (91.20%)	1464（90.7）
Complete and duplicated BUSCOs(D)	8.9%	7.6%	8.0%	8.0%	113 (7.00%)	123（7.6）
Fragmented BUSCOs(F)	0.3%	0.5%	0.1%	1.4%	18 (1.12%)	10（0.6）
Missing BUSCOs(M)	1.4%	2.5%	1.5%	0.4%	11 (0.68%)	17（1.1）
Total Lineage BUSCOs	—	—	—		1614	1614
**Assembly quality assessed by Assembly QC**						
LAI values	16.08	16.08	16.08	13.23	—	—
Sequencing depth (×)	—	—	—	Hi-C: 95	HiFi: 31.74	HiFi: 36.7

					Hi-C: 50.92	Hi-C: 165.10
Quality value (QV) from Merquury	39.74	39.74	39.74	30.01	—	—
**Gene functional annotations using different databases**						
dbCAN	1,671	1,538	1,596	1,576	—	—
EggNOG	41,143	38,192	38,955	39,865	—	33323
KEGG	20,284	19,163	19,545	19,840	—	30352
GO	29,006	27,167	27,824	27,897	—	31181
InterPro	35,255	32,777	33,548	33,240	—	—
MEROPS	1,335	1,269	1,292	1,318	—	
Pfam	28,007	26,150	27,091	27,579	—	32964
SignalP	4,143	3,778	3,952	3,860	—	
UniProt	9,517	8,988	9,234	10,783	—	
KOG_Annotation	—	—	—	—	—	22984
Swissprot_Annotation	—	—	—	—	—	28316
TrEMBL_Annotation	—	—	—	—	—	38735
Total	42,348 genes	39,147 genes	40,100 genes	72.45%(40,750 genes)	88.42 (37,366 genes)	99.32 (38,753 genes)
**Gene annotation assessment**						
Complete BUSCOs(C)	96.1%	95.1%	96.3%	—	1572 (97.40 %)	1591 (98.57%)

Complete and single-copy BUSCOs(S)	88.8%	88.6%	89.6%	—	1466（90.8%）	1472 (91.20%)
Complete and duplicated BUSCOs(D)	7.3%	6.5%	6.7%	—	106（0.07%）	119 (7.37%)
Fragmented BUSCOs(F)	1.5%	1.5%	1.4%	—	30 (1.86%)	5 (0.31%)
Missing BUSCOs(M)	2.4%	3.5%	2.3%	—	12 (0.74%)	18 (1.12%)
Total Lineage BUSCOs	—	—	—	—	1614	1614
