# Supplementary material for: Chromosome-Level Genome Assembly of Ormosia henryi Provides Insights into Evolutionary Resilience and Precision Conservation
Source: Plants (Basel). 2026 Jan 7;15(2):180. doi: 10.3390/plants15020180 (PMC12845328; doi:10.3390/plants15020180)
Supplement: Supplementary file 1 [file plants-15-00180-s001.zip › Table S9.pdf]

Table S9 Statistical information sheet of gene family clustering across ten species

Item	Adura	Ccaja	Gmax	Lalbu	Ljapo	Mtrun	Ohenr	Osati	Pvulg	Tprat
Number of genes	33,271	28,186	56,007	45,762	28,205	44,450	39,017	57,358	26,845	32,554
Number of genes in orthogroups	29,943	26,761	49,365	38,116	25,708	34,697	36,397	34,895	25,234	25,985
Number of unassigned genes	3,328	1,425	6,642	7,646	2,497	9,753	2,620	22,463	1,611	6,569
Percentage of genes in orthogroups	90	94.9	88.1	83.3	91.1	78.1	93.3	60.8	94	79.8
Percentage of unassigned genes	10	5.1	11.9	16.7	8.9	21.9	6.7	39.2	6	20.2
Number of orthogroups containing species	17,553	18,239	21,147	18,787	18,022	20,518	20,390	13,993	18,111	18,476
Percentage of orthogroups containing species	46.8	48.6	56.4	50.1	48	54.7	54.3	37.3	48.3	49.2
Number of species-specific orthogroups	856	234	975	1,270	372	1,088	427	5,384	174	464
Number of genes in species-specific orthogroups	4,976	907	2,995	6,394	1,172	3,789	2,630	23,875	677	1,224

Percentage of genes in species-specific orthogroups	15	3.2	5.3	14	4.2	8.5	6.7	41.6	2.5	3.8
